# Cross-Modal Re-Organization in Adults with Early Stage Hearing Loss

**DOI:** 10.1371/journal.pone.0090594

**Published:** 2014-02-28

**Authors:** Julia Campbell, Anu Sharma

**Affiliations:** 1 University of Colorado at Boulder, Department of Speech, Language and Hearing Sciences, Boulder, Colorado, United States of America; 2 University of Colorado at Boulder, Institute of Cognitive Science, Boulder, Colorado, United States of America; University of Modena and Reggio Emilia, Italy

## Abstract

Cortical cross-modal re-organization, or recruitment of auditory cortical areas for visual processing, has been well-documented in deafness. However, the degree of sensory deprivation necessary to induce such cortical plasticity remains unclear. We recorded visual evoked potentials (VEP) using high-density electroencephalography in nine persons with adult-onset mild-moderate hearing loss and eight normal hearing control subjects. Behavioral auditory performance was quantified using a clinical measure of speech perception-in-noise. Relative to normal hearing controls, adults with hearing loss showed significantly larger P1, N1, and P2 VEP amplitudes, decreased N1 latency, and a novel positive component (P2’) following the P2 VEP. Current source density reconstruction of VEPs revealed a shift toward ventral stream processing including activation of auditory temporal cortex in hearing-impaired adults. The hearing loss group showed worse than normal speech perception performance in noise, which was strongly correlated with a decrease in the N1 VEP latency. Overall, our findings provide the first evidence that visual cross-modal re-organization not only begins in the early stages of hearing impairment, but may also be an important factor in determining behavioral outcomes for listeners with hearing loss, a finding which demands further investigation.

## Introduction

A basic tenet of neuroplasticity is that central pathways will re-organize following long-term sensory deprivation. There is ample evidence from animal and human studies of cross-modal re-organization of the cortex that occurs in both blindness [1], [2], [3], [4], [5], [6], [7], and congenital deafness [8], [9], [10], [11], [12], [13], [14], [15], [16], [17], [18], [19], [20], [21], [22], [23]. For example, congenitally deaf white cats show enhanced motion processing and localization in the visual periphery, and recruit higher-order auditory cortex for improved performance in these tasks [19], [24]. Similarly, congenitally and post-lingually deaf humans (with and without cochlear implants) demonstrate activation of auditory cortical areas during processing of visual motion and complex visual pattern changes, which is not seen for normal hearing control subjects [15], [17], [25], [26], [27]. Although cross-modal recruitment serves to enhance behavioral performance for the recruiting modality [19], [28], it has been linked to a decrease in performance of the recruited modality. In deaf adults fitted with cochlear implants, cross-modal recruitment (measured by event-related potentials) has been correlated with decreased performance on speech perception tasks [25], [26], [29]. All of the studies mentioned above have been conducted on individuals in the most advanced stage of hearing loss (i.e., profound deafness). However, most post-lingual deafened adults show a gradual decline in hearing, which typically progresses through the mild, moderate, severe and profound stages of hearing loss [30], [31]. Thus, the degree of sensory deprivation necessary to induce cross-modal cortical plasticity remains unclear. Given the potential impact on clinical outcomes, it would be useful to determine whether cross-modal cortical changes begin during early stages of hearing decline or whether these changes are limited to the near-total sensory deprivation that accompanies deafness. In this study, we examined visual evoked potentials (VEP) using high-density electroencephalography and auditory behavioral outcome using a clinical test of speech perception in noise in persons with adult-onset mild-moderate hearing loss and normal hearing control subjects.

## Materials and Methods

### Participants and Ethics Statement

**Figure 1:**
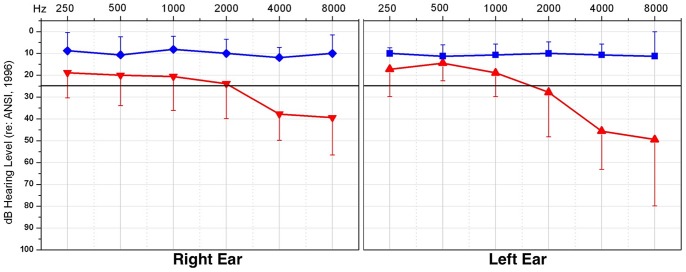
Mean audiometric subject thresholds. Auditory thresholds are shown for right and left ears for the standard audiometric frequencies from 250(NH) group are depicted in blue; the hearing loss (HL) group in red. The positive-going blue bars illustrate the standard deviation for the average threshold at the designated frequency for the NH group, and negative-going red bars illustrate the standard deviation for the HL group. The solid black line illustrates the criterion for normal hearing, at 25 dB HL.

Seventeen adults between the ages of 37 to 68 years participated in this study. The study was approved by the University of Colorado at Boulder Institutional Review Board, and all participants provided written consent. Subjects were recruited via advertisements in the community, and hearing was tested for all subjects using standard clinical audiometric procedures prior to speech-in-noise and EEG measurements. Eight of the subjects (mean age and standard deviation: 50.5+/−6.2 years; range: 37.4–57 years) revealed clinically normal hearing thresholds (i.e., below 25 dB Hearing Level) for frequencies ranging from 250 Hz to 8000 Hz. Nine of the subjects demonstrated hearing loss (mean age and standard deviation: 56.9+/−8.9 years; range: 38.4–68.2 years). On average, this group showed normal hearing from 250 Hz through 1000 Hz and a mild-to-moderate sensorineural hearing loss bilaterally from 2000 Hz to 8000 Hz. Average audiograms for the two groups are shown in Figure 1. None of the participants with hearing loss were receiving clinical intervention at the time of enrollment. However, many participants suspected a possible hearing loss prior to diagnosis. Subjects who were diagnosed with hearing loss through the study received counseling from a state-licensed clinical audiologist (first author) and referrals to audiology clinics for possible consideration of amplification. EEG testing sessions took place on separate days for those diagnosed with hearing loss unless otherwise requested. The participants in the normal hearing (NH) group and hearing loss (HL) group showed no difference in age between groups (*t*(15) = −1.69, *ρ* >0.05). All participants reported no issues with visual acuity and no neurological impairment.

### Auditory Behavioral Testing: Test of Speech Perception-in-noise

Speech perception in background noise was measured using the QuickSIN™ test [32], a clinical assessment of auditory acuity in background noise. Participants faced a speaker at 0° azimuth and were instructed to repeat two recorded sentence lists (six sentences each) presented at 65 dB Hearing Level (HL). Background noise was varied to determine the signal-to-noise ratio (SNR) required by the participant to accurately repeat 50% of the sentences. The SNR values began at 25 dB and decreased in 5 dB increments to 0 dB. The SNR score from the two lists was computed and averaged for each participant. Overall, the lower the SNR score, the better the performance on the test.

### EEG Procedures

#### Visual stimuli

Participants were shown a high contrast sinusoidal concentric grating that morphs into a radially modulated grating or circle-star pattern [25], [33], [34] on a 26-inch flat-screen LCD television at a viewing distance of approximately 42 inches. The circle and star figures were presented 150 times. The star figure was presented on the screen for 600 ms, then immediately followed by the circle figure, also lasting for 600 ms. This presentation method provided the percept of apparent motion and shape change to the viewer. A total of 300 stimulus presentations (sweeps) were presented, for a testing time of three minutes. The VEP was time-locked to the onset of each individual star and circle presentation. Participants were instructed to direct their gaze to the center of the star/circle at a black dot and to not shift gaze during the three minutes.

### EEG Recording and Analyses

Participants were fitted with a 128-channel EEG electrode recording net (Electrical Geodesic, Inc.) and seated in a comfortable reclining chair in an electro-magnetically shielded sound booth. All stimuli were presented via E-Prime® 2.0, stimulus software compatible with Net Station 4 (Electrical Geodesic, Inc). The sampling rate for the EEG recordings was 1 kHz, with a band-pass filter set at 0.1–200 Hz.

Data were band-pass filtered offline at 1–30 Hz and segmented according to the EEG activity surrounding the stimulus presentation (epochs), with 100 ms pre-stimulus and 495 ms post-stimulus time. EEG recordings were corrected to the pre-stimulus baseline, and eye-blink artifact recorded at designated eye channels was removed if greater than +/−100 µV, unless adjusted for individual subjects. Bad channels were removed from the recording and replaced with interpolated data from the remaining channels via a spline interpolation algorithm. Remaining data were averaged and re-referenced using common average reference. Individual waveform averages were averaged together for each of the two groups (i.e., the normal hearing and hearing loss group) to compute a grand-averaged waveform. Amplitudes and latencies for individual participants were recorded for all three obligatory visual evoked potential (VEP) peaks (i.e., P1, N1 and P2). The P1 peak component was observed as the first positive-going peak occurring approximately within a latency window of 90 to 130 ms, the N1 component was observed as the second peak or first negative-going peak occurring approximately between 135 ms to 200 ms, and the P2 component was observed as the third peak or second positive-going peak occurring approximately within 200 to 300 ms. If a peak component occurred outside of the described latency ranges, it was still marked and included according to the order of appearance (e.g., the first large positive component at 80 ms was marked as P1). P1 amplitudes were defined as the onset to peak value, N1 amplitudes as the peak of the N1 component to peak of the P2 component, and P2 amplitudes as peak of the P2 component to offset value. Latencies were chosen at the highest amplitude of the peak.

First we created a two-dimensional voltage map using Net Station 4 (Electrical Geodesic, Inc), which allowed us to examine regions of interest (ROI) around the occipital midline [25], [29], focusing on the greatest group differences for visual stimuli. Using planned comparisons with the Bonferroni correction, electrodes within the ROI were then chosen for statistical analysis according to the largest mean group differences for the amplitude and latency of each VEP component.

### Source Localization Analysis (Current Density Reconstructions)

EEG data for individual participants were exported from Net Station and imported into EEGLAB [35] using MatLab® (The MathWorks®, Inc., 2010). The data were corrected to the baseline of a pre-stimulus interval of 100 ms and sweeps greater than +/−100 µV were rejected as artifacts. The sampling rate was down-sampled to 250 Hz to reduce processing, altering the post-stimulus time to 492 ms. The first step in creating source models was to prune the concatenated EEG sweeps or trials for each subject through independent component analysis (ICA) [36], [37]. This statistical procedure allows for observation of the spatially fixed and temporally independent components that underlie the evoked potential [38], and is useful in precise source modeling in EEG, including for deeper generators [37], [39], [40], [41]. EEGLAB was chosen specifically for preliminary source localization analysis in order to utilize the ICA algorithm that provides for optimal cortical source localization, and to perform ICA on concatenated EEG sweeps [36], [37], [42]. Once the independent components that accounted for greatest percent variance in the evoked potential were identified in the designated timeframe for a peak component of interest (e.g., P1, N1, P2), the remaining independent components were regarded as artifact/noise and discarded. The pruned potential waveforms for each subject were then grand averaged for each group (NH and HL) and exported into CURRY® Scan 7 Neuroimaging Suite (Compumedics Neuroscan™) for source modeling. In CURRY®, an additional ICA was run on the VEP mean global field power (MGFP) (incorporating all 128 channel EEG data), with only components showing a signal-to-noise ratio (SNR) of 2.0 or greater accepted. The third VEP component in the HL group (P2’) was pruned and averaged as an individual component as it was present in a subset of this group.

Peak components for the VEP MGFP waveforms were selected separately for current density reconstruction (CDR) via sLORETA, with no a-priori restrictions placed on the model. The selected head model was standardized using the boundary element method (BEM) [43]. sLORETA, or standardized low-resolution brain electromagnetic tomography, is a specific statistical method that estimates CDR [44], [45]. The CDR is represented by a graded color scale image placed on an average MRI of 100 people. Sagittal MRI slices were selected to illustrate the greatest differences in cortical activation between the groups. Montreal Neurological Institute (MNI) co-ordinates (in millimeters) illustrate the three dimensional physical locations of each slice.

## Results

### Visual Evoked Potentials

**Figure 2:**
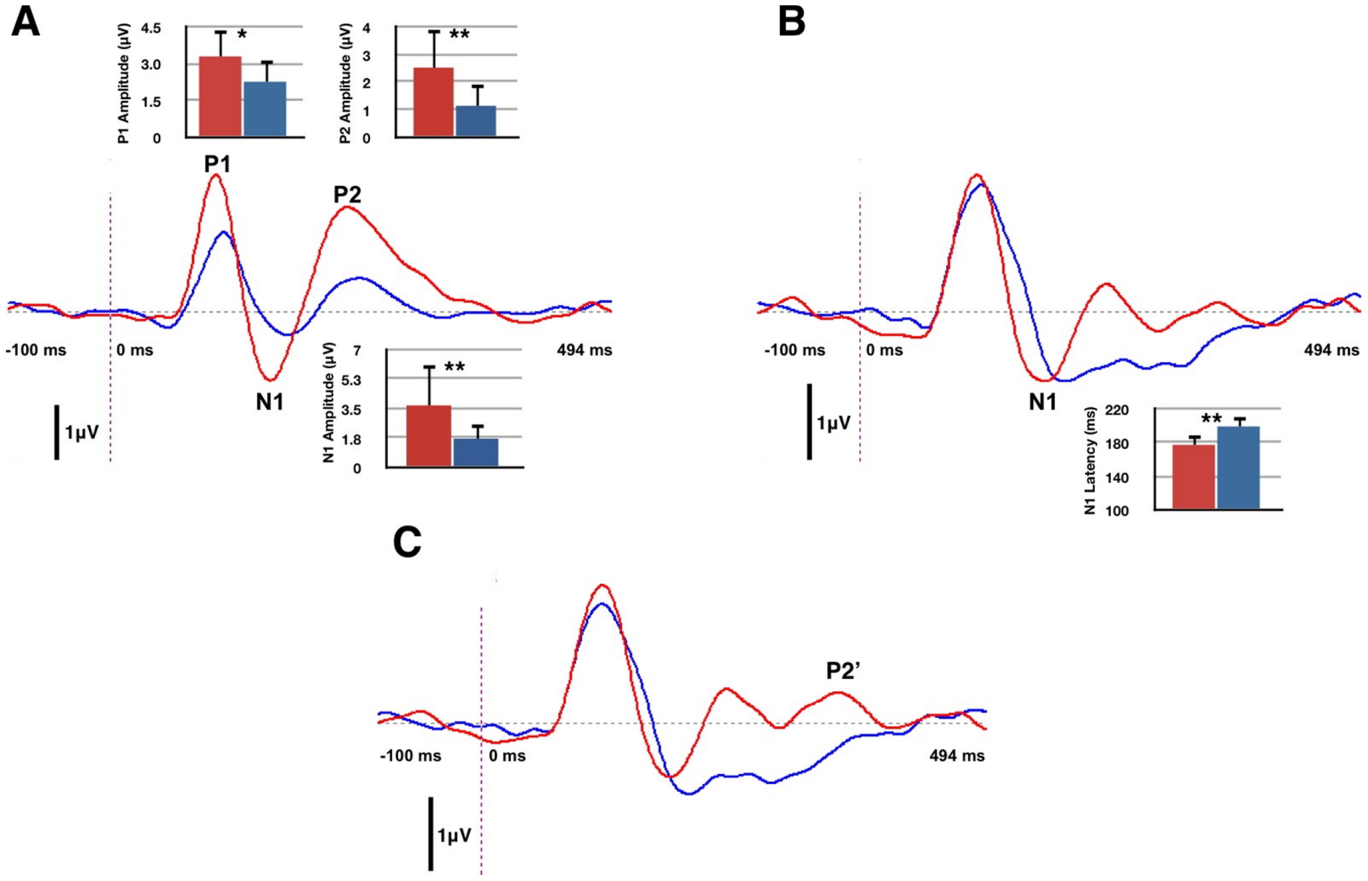
Occipital Region of Interest (ROI) cortical visual evoked potentials (VEPs). A. Peak components P1, N1, and P2 amplitudes are significantly larger for the adult Hearing Loss (HL) group (red) in comparison to the adult Normal Hearing group (blue). Mean group differences are illustrated in corresponding mean bar graphs for each component. One asterisk indicates significance at *ρ* <0.05; two asterisks indicate significance at *ρ* <0.01. B. The N1 component latency is significantly decreased in the HL group as compared to the NH group, also illustrated in the mean bar graph. C. A third positive peak component, denoted as P2’, has been found in a subset of the HL group.

Waveforms for the two groups across the whole head (128 channels) are shown in Figure 2. Three obligatory cortical VEP components elicited in response to the visual stimulus were analyzed: the P1 (occurring at approximately 100 ms), the N1 (occurring at approximately 150 ms), and the P2 (occurring at approximately 230 ms). We compared amplitude and latencies for these components between NH and HL groups at electrodes in the occipital ROI. A one-way ANOVA was computed to compare group differences. Post-hoc planned comparison of means using the Bonferroni correction was completed to describe significant differences between electrodes (see Figure 2).

P1 amplitude was larger for the HL group (*F*(1, 285) = 6.265, *ρ* <0.05). N1 amplitude was larger for the HL group (*F*(1, 285) = 9.865, *ρ* <0.01). N1 latency was decreased for the HL group (*F*(1, 285) = 7.684, *ρ* <0.01). Finally, P2 amplitudes were increased for the HL group (*F*(1, 285) = 8.983, *ρ* <0.01). Overall, this trend of decreased latencies and increased amplitudes for obligatory VEP components for HL listeners is consistent with previous results, which showed evidence of cross-modal recruitment in deaf subjects [13], [25], [26], [46].

An unexpected finding was the visual identification of a positive component following the P2 (occurring between approximately 295 and 395 ms) in the HL group (see Figure 2C). We labeled this component P2’. While a possible component similar to this is observed by Doucet and colleagues [25], it was not analyzed or discussed in that study. Figure 2 shows the evoked potential waveforms for both groups at the described electrodes, with mean bar graphs illustrating significant differences.

### Cortical Source Localization

**Figure 3:**
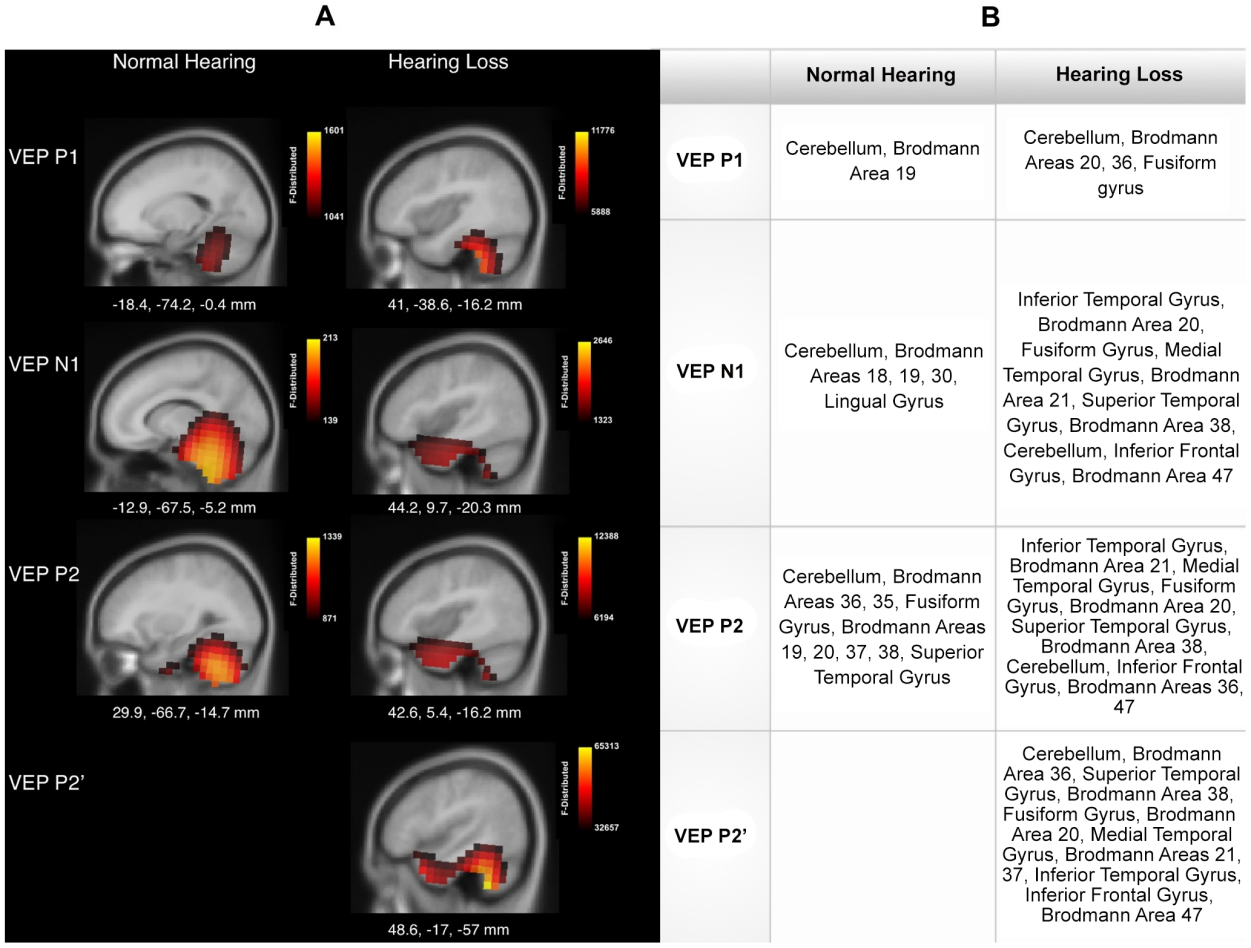
Current source density reconstructions for the NH and HL groups. A. The cortical activation at the P1, N1 and P2 VEP peak components using sagittal magnetic resonance imaging (MRI) slices. The scale of the F Distribution is shown in a scale the upper right corner ranging from red to yellow (where yellow reflects the greater strength of activation). The Montreal Neurological Institute (MNI) coordinates are listed beneath each MRI slice. B. The table describing the activated cortical regions for the VEP components for the NH and HL groups, listed in approximate order of highest level of activation.

Cortical source localization, or current density reconstruction (CDR), was performed in order to visualize anatomical regions of possible cross-modal re-organization in the HL group. The sLORETA algorithm provided by CURRY® Scan 7 Neuroimaging Suite was applied to the three VEP peak components (Figure 3). The activations were superimposed on an average MRI (sagittal slice view) and the MNI co-ordinates are shown beneath each slice. The scale of the F distribution, indicating the strength of the activations, is also shown.

As expected, for the NH group, the visual stimuli elicited all three VEP components and activated visual processing regions, including multiple cerebellar areas, which have been shown to respond to visual motion [47], [48] (Figure 3). Higher-order visual cortical regions such as Brodmann areas 18 and 19, and the fusiform region, were also activated. These findings are consistent with previous studies, which used stimuli generally similar to ours in NH subjects [34], [47], [48], [49]. The P1 component showed similar cortical and cerebellar activation for both groups. However, for the N1 and P2 components, the HL group showed greater activation along the ventral visual stream in temporal areas, which are traditionally associated with auditory processing (including superior temporal gyrus (STG), medial temporal gyrus (MTG), and inferior temporal gyrus (ITG)). This result is consistent with previous reports of cross-modal activation of temporal areas in deaf subjects [15], [17], [27]. Figure 3 shows the current density reconstructions for the NH and HL groups. A table is provided in Figure 3 describing activated regions corresponding to each of the peak components. Interestingly, the P2’ component (seen only in the HL group) showed activation of both cerebellar/occipital regions as well as temporal areas. This response pattern suggests that an additional processing step may take place within the ventral visual stream in listeners with hearing loss.

### Behavioral Performance

**Figure 4:**
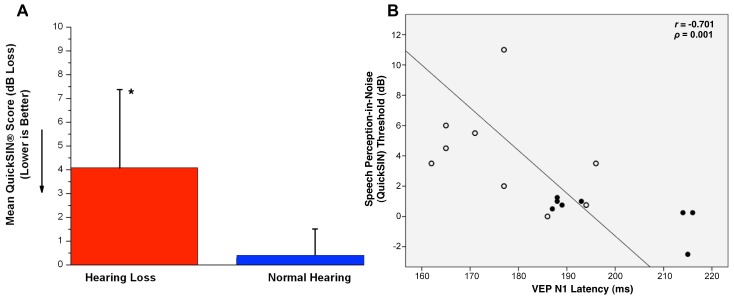
Mean scores on the QuickSIN test for the NH and HL groups, (A). Error bars are shown as vertical black lines. The asterisk reflects significant differences at *ρ* <0.05. B. QuickSIN scores are shown on the vertical axis and N1 VEP component latencies on the horizontal axis. Values are shown as closed circles for the NH group and open circles for the HL group. The Spearman’s rho value (−0.7) and significance at *ρ* = 0.001 are indicated on the upper right hand corner.

Speech perception-in-noise acuity was measured for both groups using the QuickSIN™ clinical test [32]. The results of the QuickSIN™ are reported as a signal-to-noise ratio (SNR) threshold; therefore a lower score reflects better performance. Mean scores for the NH and HL groups are shown in Figure 4A. A Mann-Whitney U Test revealed a significant difference between the two groups (*U* = 10.5, *Z* = −2.46, *ρ* <0.05). This difference in auditory performance in background noise between normal hearing listeners and listeners with mild-to-moderate hearing loss is consistent with Killion et al. [32] and Wilson et al. [50].

The N1 VEP component has been suggested as a marker of cross-modal re-organization in deafness [13], [26], [46], [51]. Therefore, we correlated the latency of the N1 with QuickSIN™ scores for the subjects. Due to hearing loss consisting of a gradual increase in auditory threshold from 0 dB HL, we included the N1 latency values and QuickSIN scores of all 17 participants in the correlation analysis. Mean QuickSIN™ scores and N1 VEP latencies were tested for normal distribution, and a Spearman’s rank-order correlation was computed due to the non-normal distribution of the data. As seen in Figure 4B, a negative correlation was observed between N1 latency and QuickSIN scores (*r* = −0.701, *ρ* = 0.001). That is, a shorter N1 latency was associated with higher scores (i.e., worse performance) on the QuickSIN™ test. Overall, our results reflecting differences in speech-in-noise perception between the NH and HL groups as a function of visual evoked potentials are consistent with previous studies in deaf subjects showing cross-modal re-organization in subjects with poor speech perception [25], [26]. N1 latency changes also showed a significant negative correlation with the pure tone threshold averages (PTA) at 500, 1000, and 2000 Hz, which is a clinically relevant indicator of audiometric function (right ear, *r* = −0.446, *ρ* <0.05; left ear, *r* = −0.540, *ρ* <0.05). That is, as the degree of hearing loss increased, there was a corresponding decrease in N1 latency.

## Discussion

We sought to examine whether cross-modal recruitment is evident in early stages of hearing decline or whether cross-modal plasticity is limited to the near-total sensory deprivation that accompanies profound deafness. We recorded high-density EEG in response to a visual stimulus in adults with normal hearing in the low frequencies and a mild-to-moderate hearing loss in the high frequencies. A group of age-matched normal hearing adults served as the control group. All participants were administered the QuickSIN, a test of speech-in-noise perception which is used to document clinical outcomes in patients with hearing loss.

Relative to normal hearing controls, adults with mild-to-moderate hearing loss showed: (i) increased amplitude of the P1, N1 and P2 VEP components, (ii) presence of an additional positive VEP component (P2’) occurring after the P2, (iii) decreased latency of the N1 VEP, (iv) cortical re-organization as evidenced by increased activation of auditory temporal areas elicited by visual stimulation, (iv) poorer speech perception scores in noise, (v) a significant negative correlation between the degree of hearing loss and N1 VEP latency, and (vi) a strong negative correlation between speech-in-noise perception and N1 VEP latency. Overall, this pattern of results in our listeners with mild hearing loss is consistent with previous findings in deaf subjects suggesting visual cross-modal recruitment in deafness [13], [25], [26], [51].

Consistent with our findings, significantly increased amplitudes of N1 and P2 VEPs with hearing loss are well-documented in deafness [13], [46], [51]. More recently, changes in visual shift of form and motion have been shown to elicit larger N1 and P2 responses correlated with poor speech perception in deaf, cochlear-implanted adults, respectively [25], [26]. Interestingly, smaller than normal amplitude of the visual P1 component has recently been reported in cochlear-implanted adults [29]. However, Sandmann and colleagues used a stimulus consisting of four separate checkerboard reversal patterns at varying luminance ratios, which is a more complex pattern of stimulation relative to the one used in this study. The checkerboard pattern is therefore more likely to tap into a different stage of visual processing than is evident in this study [29]. Typically, decreased latencies and increased amplitudes of evoked potential components are considered to be reflective of faster processing [52], [53] suggesting that HL subjects recruit additional cortical areas to subserve processing speed and/or efficiency. To this end, we identified the P2’ VEP component (following the P2) only in the HL group, possibly indicating a new or additional generator facilitating visual processing in the HL group.

Current source density reconstructions (CDR) were compared between NH and HL listeners. As expected, for the visual stimulus, NH listeners showed cerebellar/occipital activation for the P1, N1 and P2 VEP components (Figure 3). Responsive regions included Brodmann areas 18 and 19, which comprise higher-order visual cortex. Visual stimuli comparable in both shape and appearance of motion to the one used in the present study have been shown to activate similar cortical regions in VEP and fMRI imaging studies [34], [47], [54].

The HL group showed occipital/cerebellar activation comparable to the NH group, as was evident in the CDR for the P1 response. However, higher-order processing as reflected by the CDR for the N1 and P2 components showed clear evidence of cortical re-organization. Cortical activation for these components showed an emphasis in ventral stream processing within temporal cortex, including temporal gyri (ITG, MTG and STG), which are typically associated with auditory cortical processing [55], [56]. The P2’ component, identified in only the HL group, showed underlying activation of both cerebellar/occipital areas and temporal areas, suggestive of a possible new generator in temporal cortex subserving visual processing.

In a recent study, Campbell and Sharma [57] examined cortical responses to auditory stimulation in adults with mild-moderate hearing loss. The authors reported a change in cortical resource allocation, including decreased temporal activation in STG and increased frontal activation in response to passive auditory stimulation in mild-moderate hearing loss. Taken together with the present results, this suggests that a decreased temporal activation to sound in mild-moderate hearing loss may be coincidental with the increased visual activation of temporal areas in this study.

Overall, the CDR findings are strongly suggestive of cortical re-organization facilitating cross-modal recruitment for visual processing in adults with mild-moderate hearing loss. The shift of activation to temporal cortex represents activation of the ventral visual stream, which is typically responsive to visual object form or shape changes, and is located within temporal cortex in proximity to auditory areas. The ventral stream has been implicated in the processing of facial and mouth movements [58], [59]. Thus, our results may be suggestive of compensatory plasticity as HL listeners begin to rely on facial information as a strategy to compensate for their hearing impairment [60], [61], [62], [63], [64]. Because the ventral stream is largely responsible for processing object and face information, a heavier processing load may be imposed on this stream when listeners with hearing loss begin to pay more attention to lip and facial cues. Indeed, visual attention is a modulatory influence for compensatory plasticity, and congruent visual input has shown to enhance auditory speech perception performance in cochlear-implanted adults [18], [65]. A recent study by Strelnikov and colleagues suggests that increased intra-modal compensatory activity in occipital cortex predicts better outcomes for post-lingually deaf adults after cochlear implantation, presumably due to the synergy of the visual system in deciphering auditory information and ultimately increasing the ability for auditory discrimination when sound is re-introduced via implantation [65]. However, similar to the present study, increased cross-modal re-organization in superior temporal sulcus (STS) appears to predict poor outcomes for both pre- and post-lingually deaf implant users [65], [66], [67].

Hearing loss is most consistently associated with poor outcomes in recognizing speech in background noise, a skill essential for everyday listening [30], [68], [69], [70]. Consistent with previous research in hearing-impaired listeners, our results show that listeners with even mild-to-moderate hearing loss demonstrate a significant deficit when listening to speech in background noise [71], [72]. A strong negative correlation was observed between speech perception-in-noise performance on the QuickSIN test and N1 VEP latency. That is, a shorter N1 latency was associated with higher scores (i.e., worse performance) on the QuickSIN™ test. While hearing loss is a well-known contributor to decreased speech in noise performance [73], [74], our results suggest that cross-modal plasticity may also be an important factor that should be considered in the decreased auditory performance in background noise of listeners with hearing loss. If we assume that cross-modal plasticity implies a greater reliance on lip-reading, then it might possibly serve as a facilitatory compensation in noisy situations where congruent visual input enhances auditory processing [65]. A decrease in N1 latency was also correlated with higher audiometric thresholds, suggesting a possible increase in cross-modal recruitment as hearing loss gets worse. Future studies should systematically describe the extent of cross-modal recruitment as a function of hearing loss ranging from mild to profound, as well as investigate the possible contribution of cross-modal plasticity on speech perception performance.

Overall, the VEP and behavioral results that we describe are strongly indicative of visual cross-modal re-organization in adults with mild-moderate hearing loss. This is a new finding as previous reports of cross-modal plasticity have been confined to adults with deafness, which was congenital or pre-lingual, and/or in cochlear-implanted adults [13], [25], [27], [51], [65], [75], [76]. The mechanisms of cross-modal plasticity in both deafness and moderate hearing loss have been explored in animal studies. Recent studies suggest that only those cortical areas involved in the sharing of multi-modal information are recruited, and still maintain the functional specificity of the original sensory modality [19], [24]. That is, higher-order, multi-modal areas are more susceptible to recruitment when a shared modality is no longer receiving appropriate input. Furthermore, this cross-modal plasticity has been found to take place as a result of moderate hearing loss, and not just profound sensory deprivation [77]. In humans, both audition and vision share object recognition functions in the ventral stream [78], [79], and are thus primed for compensatory plasticity in hearing loss. When the listening environment becomes challenging, as in background noise, greater attention to visual objects in the form of processing of faces and lips, may facilitate auditory object recognition. Along these same lines, activation of the ventral stream in adults who have experienced late-onset blindness has been correlated with poor performance in an auditory spatial task [80]. Similarly, resting state studies of pre-lingually deaf cochlear implanted children and post-lingually deaf cochlear implanted children and adults showed ventral activation in patients who had poor speech perception outcomes [66], [67]. Thus, it appears that compensatory activation, in either modality, of the cortical auditory-visual ventral stream may be associated with poorer auditory performance.

### Summary and Conclusion

Our study provides new evidence of cross-modal cortical re-organization in adult-onset mild-moderate hearing loss. Increased amplitudes of P1, N1 and P2 VEP components, decreased N1 latency, a novel P2’ component and current source density reconstructions reflecting a ventral shift in activation were observed for adults with mild to moderate hearing loss relative to normal hearing controls. Furthermore, we observed a strong negative correlation between cross-modal re-organization (as reflected by decreased N1 latency) and speech perception in noise. Future studies are needed to outline the detailed trajectory of cross-modal changes as hearing declines from a mild hearing loss to deafness. Prospective longitudinal studies will provide important information concerning the timeline of cross-modal re-organization according to severity of hearing loss, including a quantification of the degree or severity of re-organization. In addition, such studies may indicate the effect of clinical interventions, such as amplification or cochlear implantation, in reversing cross-modal re-organization.
